# Why machine learning (ML) has failed physical activity research and how we can improve

**DOI:** 10.1136/bmjsem-2021-001259

**Published:** 2022-03-16

**Authors:** Daniel Fuller, Reed Ferber, Kevin Stanley

**Affiliations:** 1 School of Human Kinetics and Recreation, Memorial University of Newfoundland, St. John's, Newfoundland, Canada; 2 Faculty of Kinesiology, University of Calgary, Calgary, Alberta, Canada; 3 Department of Computer Science, University of Saskatchewan, Saskatoon, Saskatchewan, Canada

**Keywords:** research, accelerometer, energy expenditure, evidence-based, measurement

## Abstract

Measuring physical activity is a critical issue for our understanding of the health benefits of human movement. Machine learning (ML), using accelerometer data, has become a common way to measure physical activity. ML has failed physical activity measurement research in four important ways. First, as a field, physical activity researchers have not adopted and used principles from computer science. Benchmark datasets are common in computer science and allow the direct comparison of different ML approaches. Access to and development of benchmark datasets are critical components in advancing ML for physical activity. Second, the priority of methods development focused on ML has created blind spots in physical activity measurement. Methods, other than cut-point approaches, may be sufficient or superior to ML but these are not prioritised in our research. Third, while ML methods are common in published papers, their integration with software is rare. Physical activity researchers must continue developing and integrating ML methods into software to be fully adopted by applied researchers in the discipline. Finally, training continues to limit the uptake of ML in applied physical activity research. We must improve the development, integration and use of software that allows for ML methods’ broad training and application in the field.

**Key messages**

**What is already known**

- Physical activity measurement has important clinical consequences.
- Machine learning (ML) has become a common method for measuring physical activity.
- Disciplines outside of physical activity measurement have learned important lessons from computer science that we can take away.

**What are the new findings?**

- Benchmark datasets are an important concept that has been missing from physical activity measurement research.
- Researchers should focus on developing tools that clinicians and other researchers can use to apply new advanced methods.
- Clinicians should know the limitations of ML methods in physical activity measurement.

## Introduction

Physical activity measurement is a critical issue for our understanding of the health benefits of human movement. Accelerometers are now the standard for physical activity measurement, and machine learning (ML) is arguably the most common method for methodological advances in physical activity measurement.^1^ With the public release of the new National Health and Nutrition Examination Survey (NHANES) accelerometer data,^2^ we argue that ML has failed physical activity measurement research in four important ways: a lack of benchmark data, priority in methods development, limited software integration and absence of training. We will discuss these four points and relate them to the clinical importance of integrating the newest available methods into clinical diagnosis methods.

## Lack of benchmark data

Physical activity measurement, either in the form of activity intensity prediction or activity type prediction and the field of human activity recognition (HAR) from computer science, appears to have diverged over time. As physical activity researchers, we recently have a new journal, the *Journal of the Measurement of Human Behaviour*, dedicated to measuring human behaviour. However, we argue that as a community, we have done little to learn from and integrate the field of HAR into our work. A key concept of HAR and computer science, in general, is benchmark datasets.^3^ Benchmark datasets should have seven characteristics: relevance, representation, equity, repeatability, cost-effectiveness, scalability and transparency.^4^ Benchmark datasets, such as the WISDM V.2,^5^ are publicly available labelled datasets that provide researchers with the ability to compare different ML models. Benchmark datasets also allow for standardised and incremental improvements in algorithm performance against a common dataset. Table 1 presents a review of 17 of the commonly used benchmark datasets for HAR. On average, datasets included 24 participants (range 4–563) and there was only one benchmark dataset that included information about participant demographic characteristics,^6^ including their age, gender or mobility challenges. As with all data analyses, the quality of the underlying data is crucial for the veracity of the methods.^7^ While physical activity researchers have collected massive population-level datasets, including NHANES and the UK Biobank, there has been limited use and publication of labelled benchmark datasets. A recent systematic review included 53 studies using ML on accelerometer data and few of these studies used the same dataset.^1^ This means that for each new ML method developed, there is little or no ability to compare performance and trade-offs between these methods because the datasets are developed using different data. Moreover, physical activity researchers often prefer to collect and use their datasets for ML development, slowing the progress of methods development and limiting the ability of researchers to develop and improve on previous methods. The use of bespoke non-public datasets for training and validation also potentially compromises the generalisability of the models and findings. For example, an ML model developed for predicting physical activity types based on data from a population in London, England, may not generalise to rural Africa or even to adults in car-centric cities like Atlanta, Georgia. A focus on collecting and sharing benchmark data, combined with incremental development of new generalisable ML methods, should be a critical component in advancing this research field.

**Table 1:** Review of benchmark datasets for human activity recognition

Year	Dataset name	Demographics	Activities	Number of participants	Number of devices	Type of device	Wear location of devices	Sampling frequency	Web link
2014	User Identification From Walking Activity Data Set	No	Walking	22	1	Phone accelerometer	Chest pocket	Not mentioned	http://archive.ics.uci.edu/ml/datasets/User+Identification+From+Walking+Activity#
2012	Human Activity Recognition Using Smartphones Data Set	No	Walking	30	1	Phone accelerometer	Waist	0.3 Hz	https://archive.ics.uci.edu/ml/datasets/Human+Activity+Recognition+Using+Smartphones
2014	Dataset for ADL Recognition with Wrist-worn Accelerometer Data Set	No	14 different activities of daily living	16	1	Watch	Wrist	Not mentioned	https://archive.ics.uci.edu/ml/datasets/Dataset+for+ADL+Recognition+with+Wrist-worn+Accelerometer
2014	MHEALTH Dataset	Yes	12 different activities of daily living	10	1	ECG	1.Chest, 2. right wrist and 3. left ankle	50 Hz	http://archive.ics.uci.edu/ml/datasets/MHEALTH+Dataset
2014	REALDISP Activity Recognition Dataset	No	33 different activities of daily living	17	1	Accelerometer	Two accelerometers on each arm and leg and one on the back (nine total)	Not mentioned	http://archive.ics.uci.edu/ml/datasets/REALDISP+Activity+Recognition+Dataset
2012	OPPORTUNITY Activity Recognition Data Set	No	9 different activities of daily living	Not mentioned	3	Not mentioned		Not mentioned	http://archive.ics.uci.edu/ml/datasets/OPPORTUNITY+Activity+Recognition
2013	Activities of Daily Living (ADLs) Recognition Using Binary Sensors Data Set	No	Not mentioned	Not mentioned	1	Sensor	Not mentioned	Not mentioned	http://archive.ics.uci.edu/ml/datasets/Activities+of+Daily+Living+%28ADLs%29+Recognition+Using+Binary+Sensors
2016	Smartphone Dataset for Human Activity Recognition (HAR) in Ambient Assisted Living (AAL) Data Set	No	6 different activities of daily living	30	1	Phone	Waist	50 Hz	http://archive.ics.uci.edu/ml/datasets/Smartphone+Dataset+for+Human+Activity+Recognition+%28HAR%29+in+Ambient+Assisted+Living+%28AAL%29
2015	Smartphone-Based Recognition of Human Activities and Postural Transitions Data Set	No	6 different activities of daily living	30	1	Phone	Waist	50 Hz	http://archive.ics.uci.edu/ml/datasets/Smartphone-Based+Recognition+of+Human+Activities+and+Postural+Transitions
2012	PAMAP2 Physical Activity Monitoring Data Set	No	18 different activities of daily living	9	4	Heart rate monitor and accelerometer	1.Wrist, 2. chest and 3. dominant ankle	~9 (HR monitor) and 100 Hz (IMU)	http://archive.ics.uci.edu/ml/datasets/PAMAP2+Physical+Activity+Monitoring
2019	WISDM Smartphone and Smartwatch Activity and Biometrics Dataset	No	Not mentioned	51	2	Phone and wrist accelerometer	Not mentioned	20 Hz	http://archive.ics.uci.edu/ml/datasets/WISDM+Smartphone+and+Smartwatch+Activity+and+Biometrics+Dataset+
2014	User Identification From Walking Activity Data Set	No	Walking	22	1	Phone accelerometer	Chest pocket	Not mentioned	http://archive.ics.uci.edu/ml/datasets/User+Identification+From+Walking+Activity
2017	Performance of thigh-mounted triaxial accelerometer algorithms in objective quantification of sedentary behaviour and physical activity in older adults	No	Not mentioned	40	1	Accelerometer	Thigh	Not mentioned	https://dataverse.harvard.edu/dataset.xhtml?persistentId=doi:10.7910/DVN/QMPEI5
2015	Newcastle polysomnography and accelerometer data	No	Not mentioned	28	2	Polysomnograph and accelerometer	Wrist	Not mentioned	https://zenodo.org/record/1160410%23.X5HRnpNKg8Y
2019	Replication Data for Method to collect ground truth data for walking speed in real-world environments.	No	Walking Speed	Not mentioned	1	Accelerometer	Not mentioned	Not mentioned	https://dataverse.harvard.edu/dataset.xhtml?persistentId=doi:10.7910/DVN/QN94IG
2018	Single wrist-worn accelerometer data	No	1. Writing and 2. typing and touching (scrolling)	Not mentioned	1	Accelerometer	Wrist	Not mentioned	https://data.ncl.ac.uk/articles/Single_wrist-worn_Accelerometer_data/10281449
2020	Smartphone Gyroscope and Accelerometer Dataset for Human Activity Recognition	No	Not mentioned	4	1	Phone accelerometer	1. Front pants pocket and 2. back pants pocket	Not mentioned	https://zenodo.org/record/3925679%23.X5HSaZNKg8Y

## Priority in methods development

It has been suggested that the original cut-point measures for physical activity measurement have been left aside in favour of ML methods.^8^ While ML methods are superior to the previous cut-point-based approaches for activity intensity classification, we argue that the jump from cut-point-based approaches to ML may have missed potentially important and useful methodological advances.^1^ For example, it is plausible that advanced rule-based approaches may provide sufficiently accurate classification compared with ML methods; however, new rule-based approaches are rarely developed or compared with ML methods using benchmark data. The priority of methods development focused on ML without sufficient benchmark data has created important blind spots in physical activity measurement. Additionally, other methods from computer science could also be useful and applied to physical activity measurement. For example, the A* algorithm could impute missing data and improve efficiency when processing accelerometer data with missing values.^9^ There are likely many methods from computer science that could be applied to physical activity measurement that we are missing. As a physical activity research community, we have focused on what we believe to be state of the art ML while forgetting about many other existing methods that could be applied to physical activity measurement.

## Limited software integration

While ML methods are now common in physical activity research, their integration with commonly used software is rare. For example, both ActiLife^10^ (a stand-alone software package for analysing accelerometer data) and GGIR^11^ (an R statistical programming language package) are two commonly used accelerometer data analysis tools, yet neither apply any published ML methods and rely on arguably outdated cut-point-based algorithms. Our recent search of R packages for accelerometer data processing and physical activity measurement^12^ includes 34 packages for processing accelerometer or commercial wearable device data. This is compared with hydrology (92 R packages),^13^ psychometrics (241 R packages)^14^ and Pharmacokinetics (19 R packages).^15^ The reviewed packages suggest that few ML methods have been integrated into R packages.

Despite methods development and many publications, it is also difficult to apply these ML methods to new data, which is fundamental, one of the problems that ML is trying to solve.^7^ Notably, the Sojourn^16 17^ package does include several different ML methods for analysing Actigraph accelerometer data. Furthermore, open-source software development integration lags behind other physical activity measurement research fields. Physical activity measurement researchers must improve the integration of ML methods into packages developed for specific programming languages (eg, R or Python) and stand-alone software (eg, ActiLife). As physical activity researchers, we must continue developing and integrating new software for ML methods to be fully adopted by the discipline.

## Absence of training

Training continues to limit the uptake of ML algorithms in physical activity research. While most physical activity researchers have a strong grounding in statistical methods, few have more than a surface knowledge of ML methodology. Even when ML models are available to infer activity level, type or context, researchers have difficulty employing them as they lack expertise in data preprocessing and how to evaluate the model’s performance when applied to new data. The authors' experience working with clinical researchers running randomised controlled trials where physical activity is an outcome suggests that these researchers are reluctant to use new methods for creating an outcome variable. In contrast, they tend to use existing cut-point methods to ensure that their work is comparable across different studies. Their teams do not have the technical expertise to use these new methods to be confident in their results. As a result, new ML-based methods for calculating physical activity are slow to be integrated with clinical research and practice.

## Clinical perspective

The cut-point-derived methodology we use today has inherent errors in estimating physical activity. For example, if a device estimates a person as sufficiently active, but in reality they are not, this has important health consequences for the individual and clinical consequences for the physical activity prescription. The limitations of ML methods for physical activity prescription should be known to clinicians using these data.^18^ Knowing the limitations of specific ML methods is common in fields like radiology, where ML methods have been used for some time in clinical applications.^19 20^

## Conclusion

To improve the use of ML methods in physical activity research, we believe that as a discipline, we must use and publish benchmark datasets to allow for increased open-source methods development. We must prioritise both improvements in cut-point-based and ML methods. We must improve our development, integration and use of software that allows for the broader training and application of ML methods to advance the field of study.
